# Editing base in mouse model

**DOI:** 10.1007/s13238-017-0432-4

**Published:** 2017-06-05

**Authors:** Haoyi Wang

**Affiliations:** State Key Laboratory of Stem Cell and Reproductive Biology, Institute of Zoology, Chinese Academy of Sciences, Beijing, 100101 China

Genome editing technologies enable scientists to modify DNA sequence at specific genomic loci in various cells and species. There are several editing tools, including homing endonucleases (Silva et al., 2011), zinc finger nucleases (ZFN) (Urnov et al., 2010), transcription activator-like effector nucleases (TALENs) (Bogdanove and Voytas, 2011), and clustered regularly interspaced short palindromic repeats (CRISPR) and the CRISPR-associated proteins (Cas) system (Doudna and Charpentier, 2014). These enzymes are designed to generate DNA double strand breaks (DSBs) at specific genomic loci, which will then be repaired through either nonhomologous end joining (NHEJ) generating indel mutations, or by homology directed repair (HDR) generating pre-defined precise modification (Wiles et al., 2015).

Recently, a novel strategy was developed to generate targeted precise nucleotide changes without introducing DSBs. Ca9 protein with either one or both nuclease domains mutated (dCas9 or Cas9 nicakse) fused with cytidine deaminases was directed to specific genomic locus and replace a C with a T (or replace G with A) within a defined window (Komor et al., 2016; Nishida et al., 2016; Ma et al., 2016), Although the precision and position of base editing is variable among these studies, likely owing to the different types of cytidine deaminase domain used, this strategy is generally more efficient than HDR mediated nucleotide change. Another major advantage is that, unlike HDR that is mainly active during the G_2_/S phase, base substitution strategy is likely to be active all through the cell cycle, therefore providing an attractive tool for precise gene editing in post-mitotic cells.

Including an exciting piece of work published in this volume of *Protein and Cell*, two groups demonstrated that base editing can be applied to generate mouse models with precise modification very efficiently (Kim et al., 2017). Kim and colleagues used BE3 system, which is composed of Cas9 nickase linked to APOBEC1 domain and uracil glycosylase inhibitor (rAPOBEC1-nCas9-UGI) (Komor et al., 2016). They delivered BE3 enzyme into mouse zygotes in either mRNA or protein form by either microinjection or electroporation (Qin et al., 2015; Wang et al., 2016). Although some mice with precise C to T substitution were obtained, indel mutations were also identified quite often in these mice, potentially due to the Cas9 nickase activity (Kim et al., 2017). In comparison, Liang and colleagues took a more cautious approach. First they used BE2 system composed of dCas9, therefore will not generate DNA single strand nick. Second, they incorporated five point mutations into dCas9 to improve its specificity (Kleinstiver et al., 2016) and named the system HF2-BE2. After introducing this enzyme into mouse zygotes, efficient base editing was observed in both embryos and live born mice, consistent with Kim et al.’s results. Although dCas9 was used in this system, they still identified indel mutations in some founder mice, which is not totally surprising considering activation-induced cytidine deaminase (AID) is known to play important roles in class-switch recombination (CSR) at immunoglobulin loci (Delker et al., 2009). Unexpectedly, they found cytidine deamination outside of the sgRNA recognition sequence, albeit proximal (named as proximal-site deamination). This phenomenon has not been reported in previous studies in cell lines (Komor et al., 2016; Nishida et al., 2016; Ma et al., 2016). Comparing to plasmid transfection, microinjection will deliver much more enzyme into the zygotes, and higher enzyme activity might lead to cytidine deamination in proximal region.

These studies highlighted that base editor can serve as a powerful tool to generate precise point mutations in animal models. However, in addition to the desired precision base substitution, other types of mutations such as indel and proximal-site deamination are often generated. The next step would be to perfect the system to enable precise base editing at a single base resolution with high efficiency, without introducing extra mutations.
